# Rheology of a Nanopolymer Synthesized through Directional Assembly of DNA Nanochambers, for Magnetic Applications

**DOI:** 10.1021/acs.macromol.2c00738

**Published:** 2022-07-26

**Authors:** Deniz Mostarac, Sofia S. Kantorovich

**Affiliations:** † Faculty of Physics, University of Vienna, Boltzmanngasse 5, 1090Vienna, Austria; ‡ Research Platform MMM Mathematics-Magnetism-Materials, 1090Vienna, Austria

## Abstract

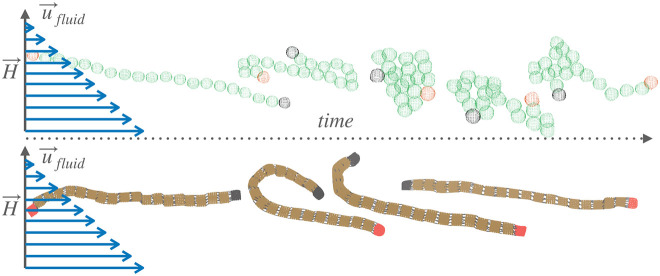

We present a numerical study of the effects of monomer shape and magnetic nature of colloids on the behavior of a single magnetic filament subjected to the simultaneous action of shear flow and a stationary external magnetic field perpendicular to the flow. We find that based on the magnetic nature of monomers, magnetic filaments exhibit a completely different phenomenology. Applying an external magnetic field strongly inhibits tumbling only for filaments with ferromagnetic monomers. Filament orientation with respect to the flow direction is in this case independent of monomer shape. In contrast, reorientational dynamics in filaments with superparamagnetic monomers are not inhibited by applied magnetic fields, but enhanced. We find that the filaments with spherical, superparamagnetic monomers, depending on the flow and external magnetic field strength, assume semipersistent, collapsed, coiled conformations, and their characteristic time of tumbling is a function of field strength. However, external magnetic fields do not affect the characteristic time of tumbling for filaments with cubic, superparamagnetic monomers, but increase how often tumbling occurs.

## Introduction

Merging polymer-like structures with magnetic nanoparticles (MNPs) is one of the ways to design magneto-responsive, soft matter systems that can capitalize on dynamic intensity control and/or great spatial resolution achievable with magnetic fields. Such systems are commonly referred to as magnetic filaments (MFs). Attempts to solve the problem of magneto-responsive material design have sparked an abundance of filament synthesis techniques^1−30^ and inspired an imposing amount of theoretical investigations.^31−50^ MFs are promising candidates for developing artificial swimmers,^51−53^ sensors,^54^ and micromixers,^55^ and they found a place in a range of applications;^56−58^ they are also used for cargo capture and transport purposes.^59,60^ Furthermore, MFs have proven useful in cellular engineering^61,62^ and designs for biomimetic cilia.^63,64^ MFs have been recognized in general as a promising system for biomedical applications.^65−67^ Magnetic fields typically do not interfere with biological tissues and processes, which makes them useful for in vivo control of engineered materials.^68^

Properties of polymer-like objects in flow are, therefore, of broad interest and great relevance in soft matter research. Polymer-like systems are known to exhibit rich and varied dynamics in shear flow. Furthermore, it is understood that their nonequilibrium conformations and reorientational dynamics can be modified in a multitude of ways apart from shear rate,^69−77^ out of which applying magnetic fields is of specific interest for this work.^78^

**Figure 1:**
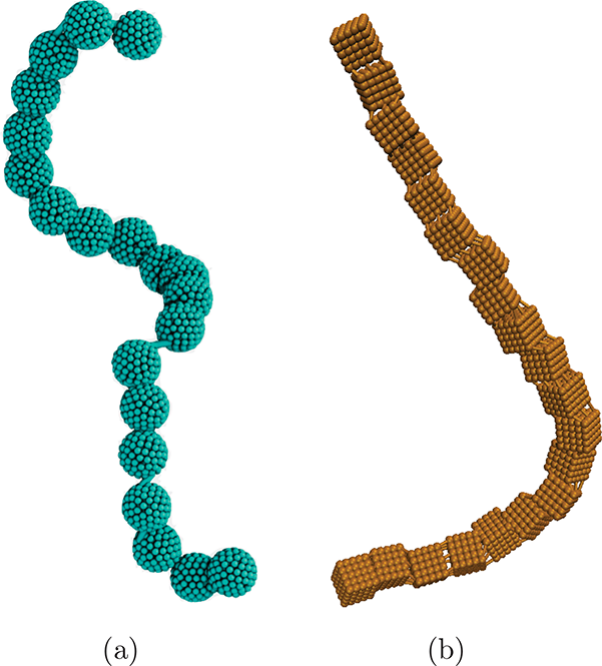
Simulation renders of models used in this work, highlighting the constrained crosslinking and raspberry monomers. (a) sMFs. (b) cMFs, based on DNC nanopolymers.

The effects of external magnetic fields on the conformational phase space available for a single magnetic filament in shear flow have hardly been explored. Even less understood are the implications of filament architecture and monomer properties, such as their magnetic nature and shape. The aim of this work is to, using Molecular dynamics (MD) simulations coupled with the Lattice-Boltzmann method, elucidate exactly these questions. Computational models we use, namely, sMFs that have spherical monomers and cMFs that have cubic monomers, are visualized in Figure 1. These models allow us to, in conjunction with varying monomer shape, consider monomers that represent two classes of MNPs, namely, magnetizable, superparamagnetic MNPs and ferromagnetic ones. Design criteria and the resulting specificities in the models shown in Figure 1 are well founded in our previous works.^79,80^ While we do not go in depth discussing the aforementioned works here, we summarize key findings and relate how are they reflected in this investigation. Detailed discussion of the implementation, parameters, and units can be found in the Simulation Methods section.

It was recently shown that divalent cuboid DNA nanochambers (DNCs) can form nanopolymers^81^ and can be used as templates for targeted assembly of nanoparticles.^82^ Contrasting filament designs based on monomer shape is inspired by this development. Polymer-like structures based on DNCs have cubic monomer shape and have highly versatile and tunable crosslinking. The magnetic response of MFs with varying magnetic nature of monomers can be remarkably similar, depending on the crosslinking. The main distinction in magnetization we found is that MFs with superparamagnetic monomers are slightly more responsive to weak magnetic fields than their counterparts with ferromagnetic monomers. However, the magnetic nature of monomers leads to vastly different filament conformations. Filaments with superparamagnetic monomers tend to bend their backbone in attempts to minimize dipole–dipole interaction energy, in external magnetic fields. This is a consequence of the magnetization effects of the dipole fields created by the MNPs. Given the existence of these local energy minima, it is reasonable to expect a different rheological response for MFs with superparamagnetic monomers than for their counterparts with ferromagnetic monomers.

One cannot, however, discuss the magnetic nature of monomers within a filament separately from crosslinking. If the rotational motion of monomers within a filament is decoupled from the backbone, magnetic properties of MFs are essentially indistinguishable based on the magnetic nature of monomers. In other words, MFs with ferromagnetic monomers look like they are MFs with superparamagnetic ones. To capture the nature of ferromagnetic MNPs in a filament, it is necessary to crosslink monomers so that their dipole moments point in the same direction along the backbone, and that their translational and rotational degrees of freedom are coupled to it. In general, monomer shape does not matter if crosslinking is restrictive enough, meaning that very short crosslinkers or very rigid ones conceal monomer shape effects. In this case, crosslinking dominates interparticle correlations. Instead, shape effects are maximized for MFs with relatively long and stretchy bonds, crosslinked in such a way that translational and rotational degrees of freedom of monomers are coupled to the backbone. The crosslinking in models shown in Figure 1 is realized so that these requirements are satisfied and referred to as constrained crosslinking, in line with the nomenclature established in Mostarac et al.^79^

In summary, sMFs stands for filaments with spherical monomers and constrained crosslinking. With this model, we build upon a typical theoretical representation of a polymer-like entity, with design choices that maximize its magnetic response. Conversely, cMFs stands for filaments, inspired by DNC nanopolymers, that have cubic monomers and a backbone that fulfills the criteria of constrained crosslinking.

## Results

Shear flow is typically characterized by the Weissenberg number, *W*. The most commonly observed reorientational behavior of polymer-like structures at high *W* (i.e., when the characteristic time of the flow is shorter than the longest molecular relaxation time) is tumbling. It is characterized by a polymer-like structure alternatively adapting stretched and collapsed conformations along the flow direction. In time, a tumbling polymer flips “head” over “tail”. It is known that, due to a competition of dipole torques caused by Zeeman coupling, and hydrodynamic torques due to the flow, a filament with ferromagnetic spherical monomers will be stabilized so that its principal axis is forming a certain angle with the flow direction.^83^ Monomer shape effects on these conclusions are, however, unknown. If rotational diffusion is important, like it is for monomers that are anisotropic due to shape and/or due to the presence of dipole moments, the model used in Lüsebrink et al.^83^ is not applicable. Here, we put forward an approach where the translational and rotational diffusion of monomers is simulated accurately.

### MFs with Ferromagnetic Monomers

**Figure 2:**
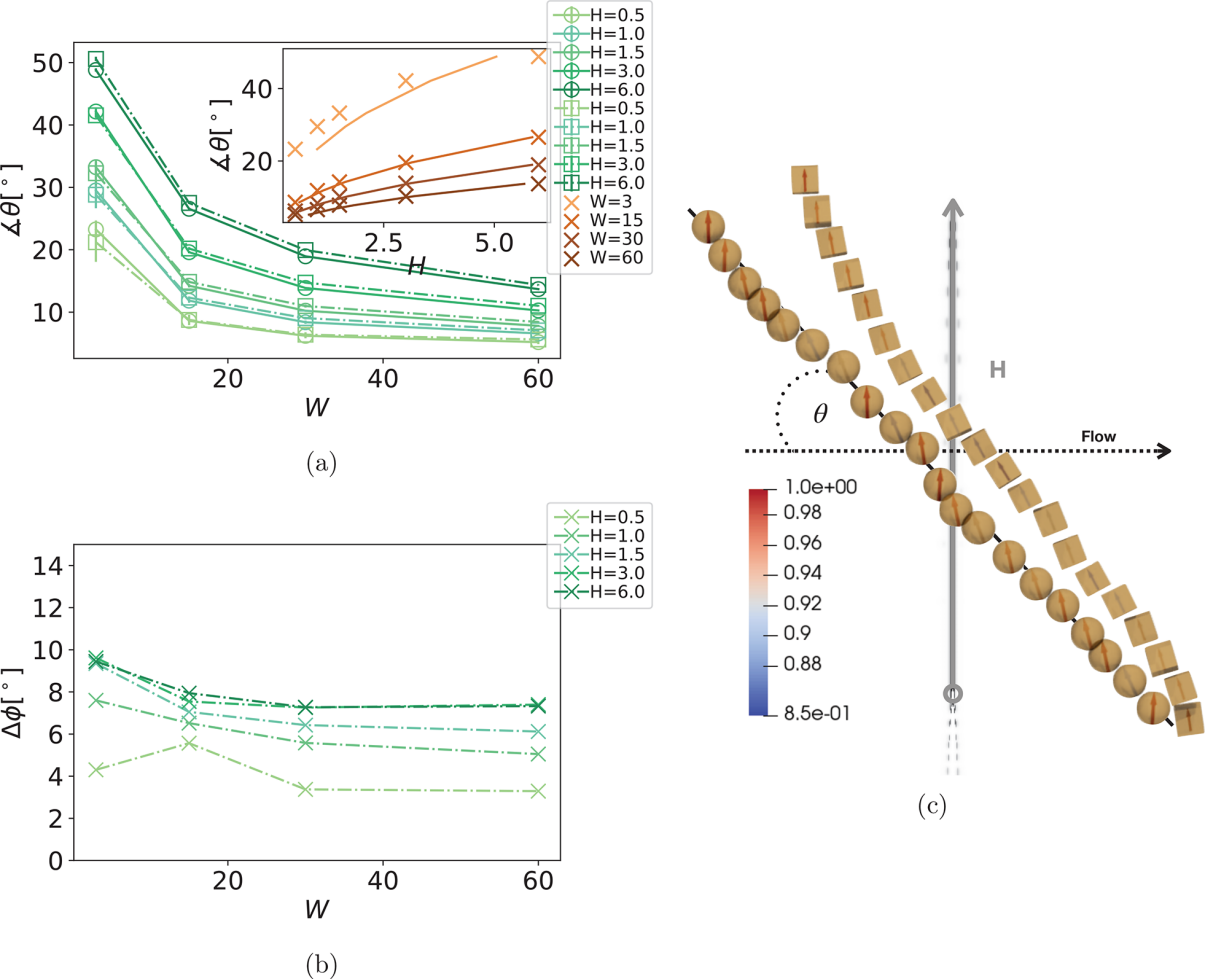
(a) Angle θ, for different *H*, as a function of *W*, comparing sMFs and cMFs. Symbol shape corresponds to monomer shape. Error bars are calculated as the standard deviation of θ, across independent runs. Inset shows θ as a function of *H*, for different filament designs and *W*. Data points correspond to simulation results, while the lines correspond to the fit of the *H* = (*a*/*b*) *sin*(θ) *tan*(θ) solution to the analytical estimation introduced in Lüsebrink et al.,^83^ where *b* cos(θ) ≠ 0. (b) Difference in ϕ between sMFs and cMFs, denoted as Δϕ, as a function of *W*, for different *H*. (c) Conformation snapshots with monomer dipole moments, corresponding to the (*W* = 3; *H* = 6) parameter set, which is the point of largest Δϕ, presented in (b). Here, we also annotate magnetic field applied, flow direction, and the angle θ used in (a) and (b). All subfigures show results for filaments with ferromagnetic monomers.

In this subsection we compare the behavior of sMFs and cMFs in case their monomers are ferromagnetic. In Figure 2a, we show the alignment angle θ between the filament main axis and the flow direction, in the flow-field plane, as a function of *W* and field strength . The orientation of the filament main axis is calculated as the eigenvector corresponding to the largest eigenvalue of the gyration tensor:
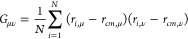
1where *r*_*i*,μ_ and *r*_*cm*,μ_ are the μ-th Cartesian component of the position of the *i*-th monomer and the center of mass, respectively.

One needs only a weak magnetic field to eliminate tumbling in MFs with ferromagnetic monomers. Looking at Figure 2a, we see that θ, as a function of *W* and *H*, is independent of monomer shape, for MFs with ferromagnetic monomers. For a fixed shear rate, increasing *H* leads to an increase in θ. Conversely, while keeping *H* fixed, an increase in *W* leads to a decrease in θ. The analytical estimation of θ obtained by balancing the hydrodynamic and magnetic torques acting on the center of mass of a filament, introduced in Lüsebrink et al.,^83^ can be used to fit our data, albeit less successfully, as shown in the inset of Figure 2a. This analytical estimation applies only if the stabilized filament conformations can be described as rod-like and if *H* and *W* are high enough to minimize thermal fluctuation effects. The limits of applicability are depicted in the fits for *W* = 3 (weak shear) and/or *H* = 6 (strong field). It comes as a surprise that monomer shape has no effect on θ. It is known that hydrodynamic forces excreted on cubic monomers are higher than for a corresponding sphere.^84^ By proxy, the overall hydrodynamic torque for cMFs is expected to be higher than that for sMFs. It must be that the increased hydrodynamic torque due to monomer cubicity is balanced with a complementary increase in magnetic torque in MFs with cubic monomers.

In Figure 2b we plot the difference in ϕ between sMFs and cMFs, denoted as Δϕ, where ϕ is the angle the overall magnetic moment of a filament , where  is the dipole moment of the *i*-th monomer and *N* is the monomer number, enclosed with the filament main axis. Indeed, the difference in hydrodynamic interactions based on monomer shape is compensated with magnetic torques. In Figure 2b, Δϕ suggests that  for sMFs is on average less aligned with the filament backbone compared to cMFs. For the parameters we explored, Δϕ is 10° at worst and 5° at best. The combination of low *W* and high *H* offers the least hydrodynamic counter-torque for dipole moments to reorient along *H⃗*. Spherical monomers easily slide past one another and rotate with respect to each other. Cubic monomer shape, on the other hand, penalizes such motion. As a result, dipole moments in cMFs are more aligned with the backbone than they are in sMFs. Therefore, magnetic torque due to Zeeman coupling is higher for MFs with cubic monomers than for their counterparts with spherical ones, and the characteristic angle θ seems to be unaffected by monomer shape. In Figure 2c we show the simulation rendered for the parameter set (*W* = 3; *H* = 6), corresponding to the highest Δϕ shown in Figure 2b, that depicts the conclusions from the paragraph above: dipole moments in an sMF fluctuate more with respect to the main axis (the deviation is color-coded, as shown on the side). Here, it can also be seen that a cMF is on average slightly more aligned with *H⃗* than its counterpart with spherical monomers.

**Figure 3:**
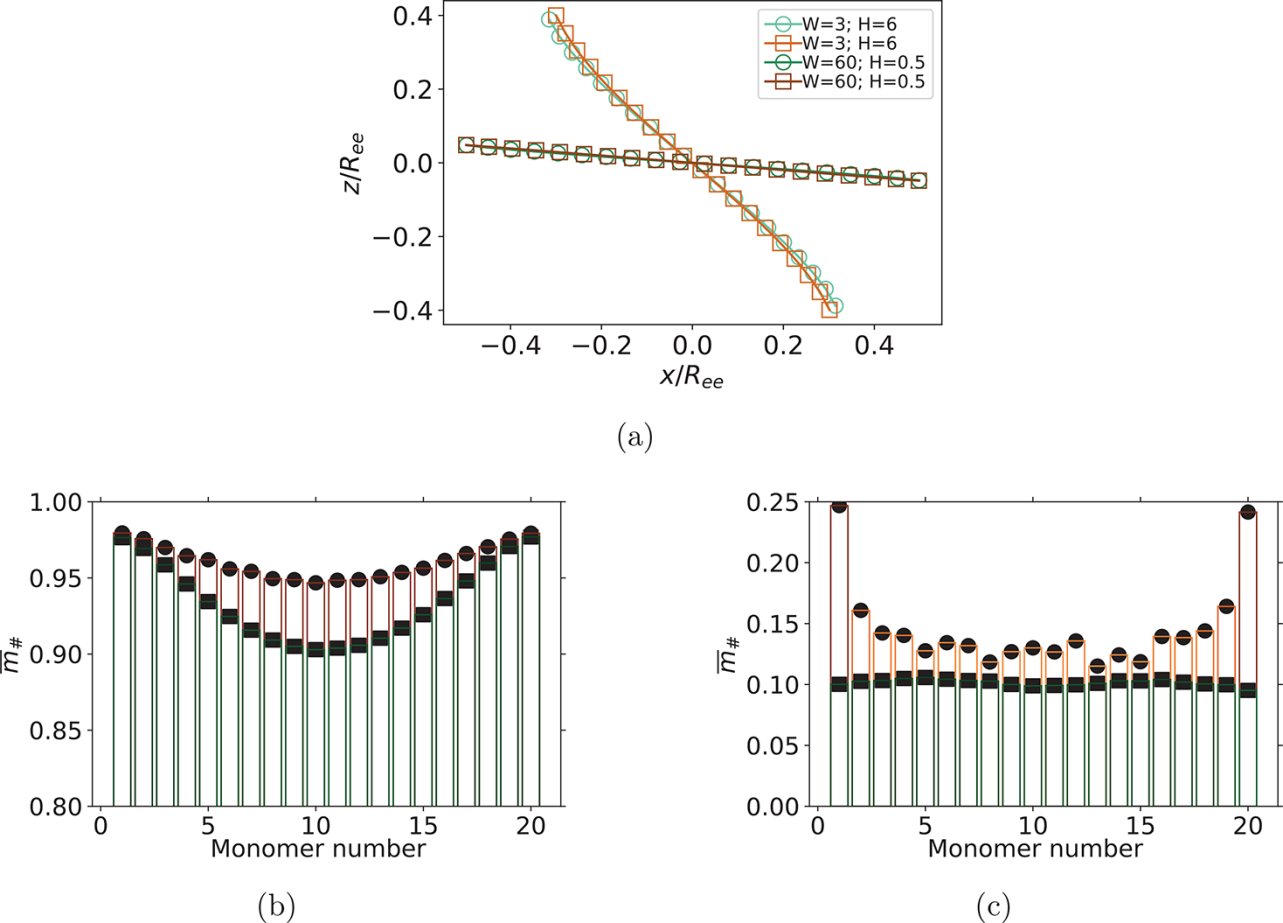
Comparison between sMFs and cMFs with ferromagnetic monomers, where (a) is showing characteristic sMF and cMF conformations, for *W* and *H* set corresponding to the largest (*W* = 3; *H* = 6) and smallest (*W* = 60; *H* = 0.5) Δϕ in Figure 2b; (b and c) Bar-plot comparison of per-particle magnetization  for (*W* = 3; *H* = 6) and (*W* = 60; *H* = 0.5) parameter sets, respectively. Characteristic conformations obtained by averaging over all simulations and snapshots, where the center of mass of each conformation was shifted to the coordinate system origin. Axes in (a) are normalized by the end-to-end distance *R*_*ee*_ for each conformation, respectively. Symbol shape corresponds to monomer shape.

Furthermore, as is shown in Figure 3a, given the interplay of magnetic and hydrodynamics torques, monomer shape, and crosslinking, there is a difference in the characteristic, stable conformations between sMFs and cMFs with ferromagnetic monomers. The conformations shown correspond to the points of largest (*W* = 3, *H* = 6) and smallest (*W* = 60, *H* = 0.5) Δϕ, shown in Figure 2b. Higher magnetic torques due to Zeeman coupling, together with increased correlations between monomer orientations due to the steric constrains inherent to cuboid shape, lead to , on average, more *S*-shaped conformations of cMFs than sMFs.

The importance of monomer shape is also well captured in Figure 3b and 3c, where we show a bar-plot comparison of per-particle magnetization , corresponding to the point of largest (*W* = 3, *H* = 6) and smallest (*W* = 60, *H* = 0.5) Δϕ, respectively. Dipole orientations are strongly correlated with the filament backbone and much more homogeneous for cMFs than for sMFs. As Zeeman coupling is competing with shear, for MFs with cubic monomers, there is an additional struggle against the steric constraints of cuboid shape. Therefore, dipole moments in the middle of the chain are aligned with the filament backbone. On the other hand, dipole moments toward the ends of the chain are more aligned with *H⃗*. Cubic monomer shape exacerbates this.

### Filaments with Superparamagnetic Monomers

While a single filament with ferromagnetic monomers, in an applied magnetic field perpendicular to the flow direction, stabilizes with a given alignment angle θ with respect to the flow direction, where θ is a function of *H* and *W*, MFs with superparamagnetic monomers exhibit far more interesting behavior. Superparamagnetic MNPs, as opposed to ferromagnetic ones, have an internal relaxation mechanism and are magnetizable by both applied magnetic fields and the dipolar fields of other MNPs surrounding them. As a result, MFs with superparamagnetic monomers access and persist in conformations impossible for their counterparts with ferromagnetic monomers.

**Figure 4:**
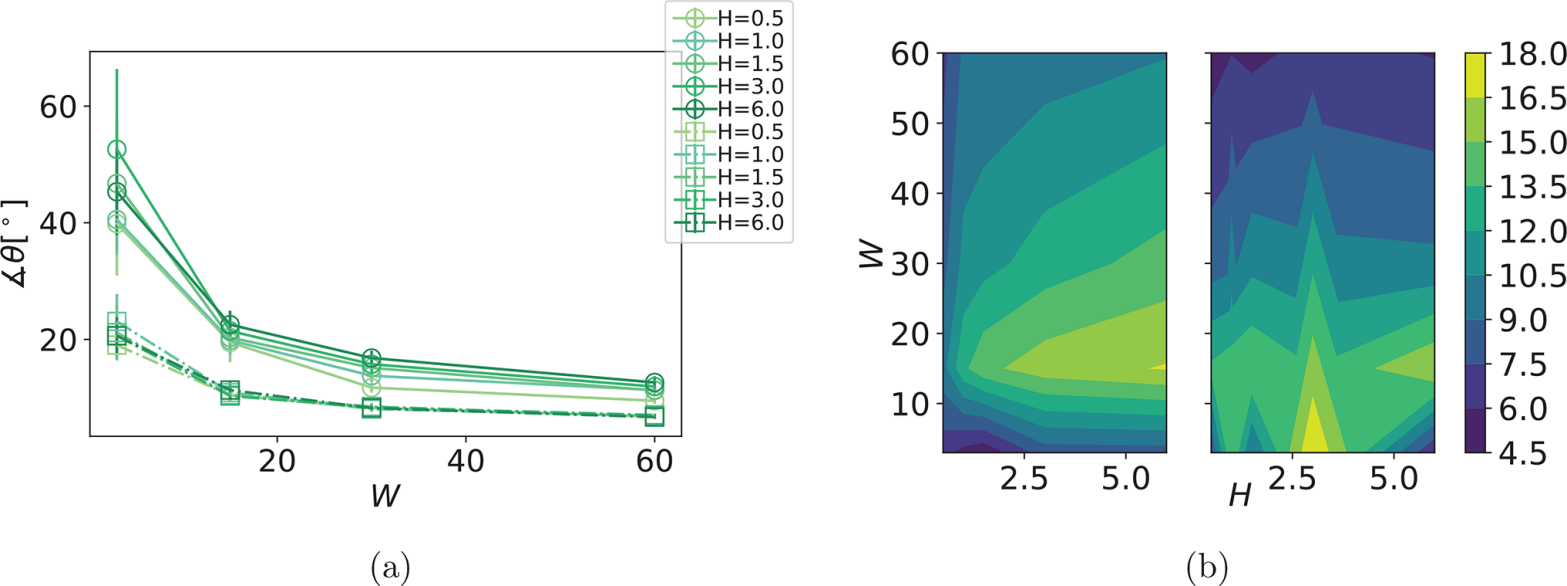
(a) Comparison in angle θ between sMFs and cMFs with superparamagnetic monomers, for different *H*, as a function of *W*. Symbol shape corresponds to monomer shape. Error bars are calculated as the standard deviation of θ, across independent runs. (b) Contour plots of the variance in θ. The contour plot on the left shows results for sMFs, while the contour plot on the right shows results for cMFs.

We start the analysis looking at Figure 4a, analogous to Figure 2a. Overall, θ(*W*) curves are like the ones seen for MFs with ferromagnetic monomers. However, they are grouped by monomer shape, rather than by field strength. We see larger θ, for sMFs than cMFs, across the *W* range we explored. Furthermore, θ drops precipitously with increasing *W*, where sMFs seem to reach a plateau around θ = 20°, while cMFs essentially align the backbone with the flow direction. The error bars in Figure 4a suggest that the averages presented are representative. However, it is revealing to consider the variance in θ, as a function of *H* and/or *W*, shown in Figure 4b.

For a given monomer shape, θ does not scale with *H*, but its variance does. sMFs with superparamagnetic monomers vary more in θ than cMFs, across the range or parameters we explored. In the *W* < 15 (low shear) region, increasing *H* decreases the variance in θ slightly. This region coincides with the highest θ in Figure 4a, and wider error bars than for any other parameter set. For low *W* and *H*, we can attribute much of the variance to thermal fluctuations. As Zeeman coupling becomes stronger with increasing *H*, we enter the range where shear forces are low enough that we see MFs assuming relatively persistent conformations. However, MFs with superparamagnetic monomers tend to bend the backbone as they try to align themselves along *H⃗* and as a result of this, they get stuck for a period of time in rather distinct conformations that correspond to local energy minima.^79^ This explains the wide error bars in Figure 4a that we see for *W* = 3. Increasing *H* tends to increase the cost of leaving such local minima in the conformational spectrum, which is why we see a decrease in variance. However, out of the *W* < 15 region, we see inverse trends. Hydrodynamic torques become strong enough to compete with magnetic torques, and filaments enter the buckling regime. Variance decreases with increasing *W*, as the hydrodynamic interactions tend to extend the filament along the flow direction. Meanwhile, magnetic dipoles attempt to establish a favorable configuration along *H⃗*, and due to Zeeman coupling, variance increases with increasing *H*. For cMFs with superparamagnetic monomers, while we see a similar trend, where an increase in *W* decreases variance, we do not capture a systematic scaling with *H*. The inspection of variance in θ in Figure 4b shows that a single filament with superparamagnetic monomers rotates in shear flow even when exposed to an external magnetic field perpendicular to the flow direction, and it does so with important differences based on its monomer shape.

**Figure 5:**
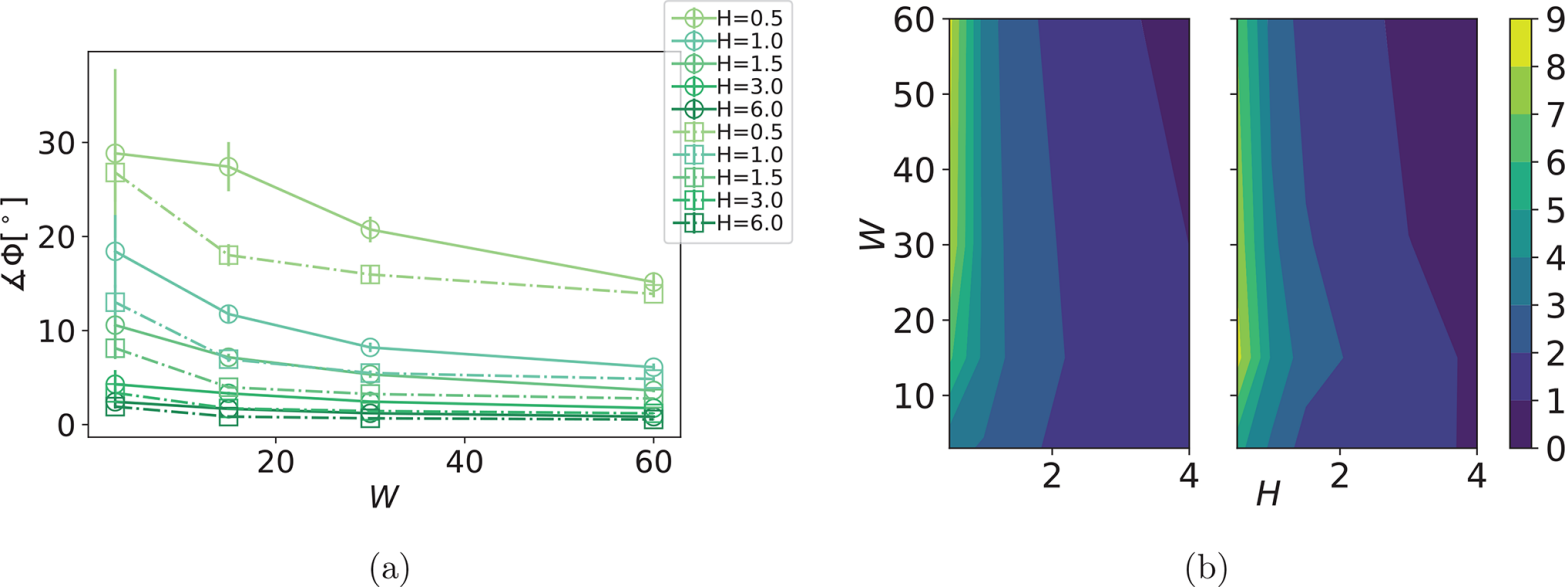
(a) Angle Φ as a function of *W*, and its scaling with field strength *H*, for MFs with superparamagnetic monomers. Symbol shape corresponds to monomer shape. Error bars are calculated as the standard deviation of Φ, across independent runs. (b) Contour plots of the variance in Φ. The contour plot on the left shows results for sMFs, while the contour plot on the right shows results for cMFs.

In order to gain deeper insight into the reason for this, we consider the angle between  and *H⃗*, denoted as Φ, as a function of *W* and *H*, shown in Figure 5a. An idealized representation of superparamagnetic MNPs, where one discounts the magnetization effects of dipolar fields, would have dipole moments fully aligned with *H⃗*.

Looking at Figure 5a,  in cMFs is more aligned with *H⃗* than in sMFs. The difference in Φ decreases with increasing *W*. Interestingly, an increase in *W* helps  to align with *H⃗*, which is initially rather counterintuitive. However, in relation with Figure 4b, it can be understood that high *W* forces MFs into conformations aligned with the flow direction and decreases variance in Φ. The flow profile constrains the translational motion of monomers to a plane and minimizes variance in the normal direction. As a result, dipole field configuration is more homogeneous, and  points along *H⃗* more. In the case of cMFs, the additional steric restrictions, compared to sMFs, reduce dipole field fluctuations even more, which is why we see that  of cMFs is overall more aligned with *H⃗*. This is corroborated with the variance in Φ, shown in Figure 5b. Overall, magnetic moment orientation is quite robust in time, and we see significant variance only for low *H*. It takes a moderate magnetic field to constrain Φ within 5°. Analogously, we have seen in Figure 4b that cubic monomer shape also leads to less overall variance in θ. With increasing Zeeman coupling, we can also see a damping effect on the variance in Φ with increasing *W*.

Summarizing the discussion above, we have seen that a single filament with superparamagnetic monomers in low shear can, regardless of monomer shape, assume semipersistent, bent conformations. The free energy landscape of MFs with superparamagnetic monomers is populated with local energy minima, corresponding to bent backbone states. However, with increasing *W*, MFs with superparamagnetic monomers start to rotate. Filaments with cubic monomers are mostly aligned with the flow direction, while their counterparts with spherical monomers are not, even for the highest *W* we explored. Furthermore, the variance in the backbone orientation with respect to the flow direction increases with *H* for sMFs, which suggests that the conformational variety correlates with *H*. For cMFs this does not seem to be the case. Lastly, magnetic moments in cMFs with superparamagnetic monomers are more aligned with the external field direction than in sMFs. An increase of *H* and/or *W* aligns  more with *H⃗* and reduces variance, regardless of monomer shape. We can infer that the reorientational mechanism of a single filament with cubic, superparamagnetic monomers in shear flow and a magnetic field applied perpendicular to the flow direction must look quite different from what it looks like for its counterpart with spherical monomers.

**Figure 6:**
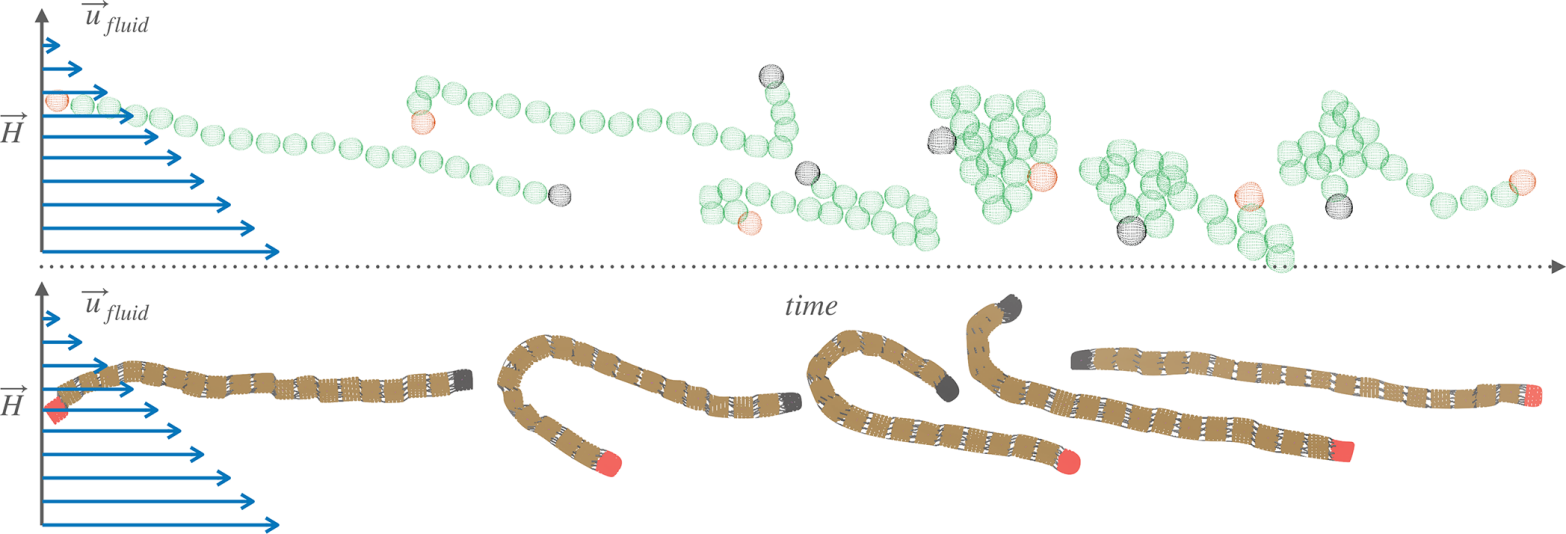
Simulation snapshots that capture the reorientational dynamics exhibited by sMFs (up) and cMFs (down) with superparamagnetic monomers, in shear flow *W* = 30 and an external magnetic field applied perpendicular to the flow direction, with a magnitude *H* = 3.0. First and last monomer of the conformations shown are colored red and black respectively, to help track their position.

In Figure 6, it can be seen that due to the internal relaxation and magnetization of superparamagnetic monomers, sMFs assume bent, coiled up, and collapsed conformations. Cuboid monomer shape instead restricts the phase-space of accessible conformations and stops the backbone from collapsing. Conformations depicted in Figure 6 all fall under the definition of tumbling. However, these are vastly different kinds of tumbling. The collapsed conformations we see for sMFs are held together by both entropy and magnetic interactions. Therefore, such collapsed conformations are not analogous to entropic coiling as a part of tumbling.

Having seen that a filament with superparamagnetic monomers tumbles in shear flow even in external magnetic fields applied perpendicular to the flow direction, we characterize the effects of monomer shape on tumbling using the diagonal elements of the gyration tensor to construct a cross-correlation function in the flow-field plane *C*_*xz*_(*t*),
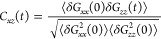
where *δG*_*ab*_ = *G*_*ab*_ – ⟨*G*_*ab*_⟩ is the component-wise fluctuation of *G*_*ab*_ around its mean. Generally, polymers and polymer-like systems in shear flow expand in the flow direction, with their motion mostly constrained in the flow-vorticity plane. However, this is entropically rather unfavorable. Thermal fluctuations lead to a stochastic extension of the polymer normal to the flow-vorticity plane, where due to the flow profile the polymer experiences torque. As a result, the polymer tumbles. Tumbling dynamics are characterized by a strong anticorrelation peak in the *C*_*xz*_ and a correlation peak for negative lags. Given the stochastic nature of the process outlined above, *C*_*xz*_ correlations decay very quickly, implying that tumbling dynamics are cyclic rather than periodic. Filaments with superparamagnetic monomers, however, have Zeeman coupling as an additional driving mechanism.

**Figure 7:**
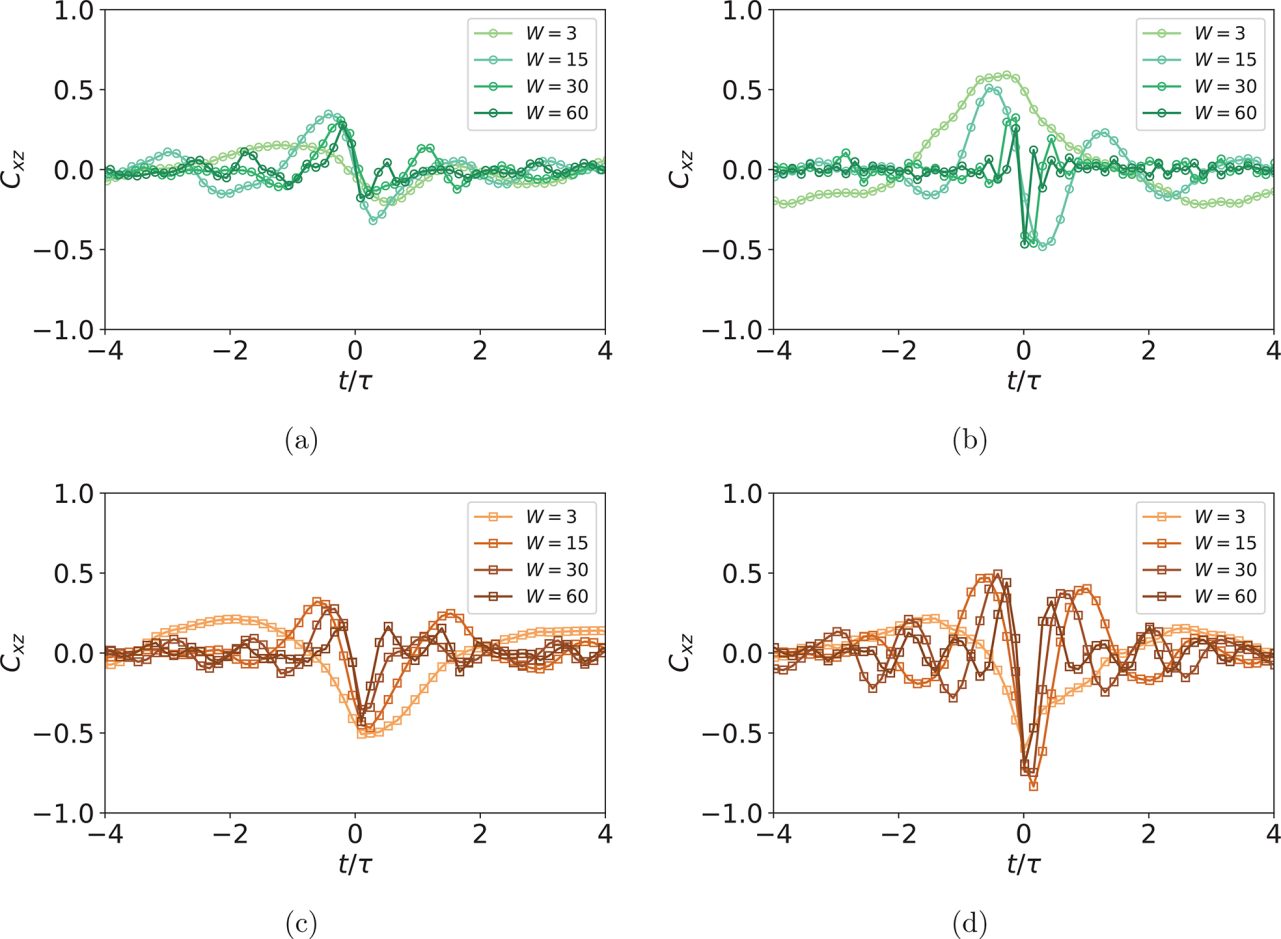
Showing a comparison of *C*_*xz*_ for sMFs and cMFs with superparamagnetic monomers, with and without an external magnetic field applied (*H* = 0 or *H* = 6), at different shear rates *W* ∈ {3, 15, 30, 60}. Time is normalized by the characteristic relaxation time τ for each filament model, defined as the time it takes for the autocorrelation function of the radius of gyration to decay in a thermalized fluid. Symbol shape corresponds to monomer shape. (a and b) Results for sMFs; (c and d) results for cMFs. (a and c) Results for *H* = 0; (b and d) results for *H* = 6.

Looking at Figure 7a, we see a typical *C*_*xz*_ of a polymer, as superparamagnetic monomers have no remanent magnetization without an external magnetic field applied. We see that, at *H* = 0, sMFs with superparamagnetic monomers tumble in a cyclic fashion, with a characteristic time of tumbling τ_*tb*_ ∝ *W*^–2/3^, as for a linear polymer.^85^ The anticorrelation peak signifies that a contraction along the flow direction is related to an extension in the field direction, and vice versa. In other words, as the chain tumbles, it coils. The peak for negative lags suggests that collapsed states along the flow are correlated with previous collapsed states along *H⃗*. However, once we turn on a strong external magnetic field, *H* = 6, perpendicular to the flow direction, the differences are profound. While MFs with ferromagnetic monomers follow the flow with a fixed orientation with respect to *H⃗*, irrespective of monomer shape, MFs with superparamagnetic monomers continue to tumble. As it can be seen in Figure 7b, the slowly decaying anticorrelation peak in low shear (*W* = 3) shifts several characteristic filament relaxation times τ. As we increase *W*, we reproduce *C*_*xz*_ profiles that signal tumbling. However, τ_*tb*_ does not scale with *W* as it did for *H* = 0, and the high *W* cross-correlation functions seem to be damped. As we have previously stated, MFs with superparamagnetic monomers tend to bend their backbone as they align with *H⃗*, instead of rotating as a whole. For low shear, orientation of MFs is mostly determined by Zeeman coupling, and it can be understood that, instead of reorientation, sMFs bend and follow the flow in bent conformations. Therefore, in addition to a shifted anticorrelation peak, we see a slowly decaying, pronounced correlation peak for negative lags. With increasing *W*, hydrodynamic forces become strong enough that conformations enforced by Zeeman coupling cannot persist, and tumbling occurs. However, for high *W*, the filament backbone collapses into coiled conformations, as depicted in Figure 6. These conformations are held together by magnetic interactions in addition to entropy, and they eventually extend along the flow direction and collapse again. This would not be possible to resolve without proper consideration of the magnetization of superparamagnetic monomers induced by the dipole fields, as such conformations would be magnetically extremely unfavorable, and the chain would break apart. Furthermore, coiled conformations we observe have dynamics of their own. They rotate as a whole and slowly unwind themselves as shear tries to break the globule apart. Therefore, we see damped *C*_*xz*_ profiles, as τ_*tb*_ is influenced by eigenmodes of oscillation of the coiled structure, which correspond to much higher frequencies. In fact, fitting the region 30 ≤ *W* ≤ 60 with a power law we get τ_*tb*_ ∝ *W*^–1.1^.

Looking at *C*_*xz*_ profiles for cMFs without an external magnetic field applied, shown in Figure 7c, it is apparent that monomer shape has a tremendous impact on the dynamics of MFs in shear flow. For low shear *W* = 3, *C*_*xz*_ does not suggest that there is tumbling. Steric constrains imposed by cubic monomer shape inhibit coiling and tumbling. Therefore, τ_*tb*_ is shifted outside of our simulation window. With increasing *W*, we start to see tumbling again, but with an overall longer τ_*tb*_ than we saw for sMFs. Furthermore, we see that the *C*_*xz*_ profiles are overall more symmetric. This is interesting, as the symmetry of the profile can reveal how the tumbling looks like. As stated before, a correlation peak for negative lags says that a collapsed state along the flow direction is correlated with a previous collapsed state along the field direction. A positive peak for positive lags, on the other hand, signifies that a collapsed state along the flow direction is correlated to a future collapsed state along field direction. For MFs with spherical monomers, the asymmetry between the correlation peaks for positive and negative lags is there since during tumbling, once the filament coils, it tends to stay coiled for some time. However, cubic monomers make coiling difficult. Instead, cMFs tumble in a distinct bend and flip motion, as depicted Figure 6. Therefore, correlation peaks for both positive and negative lags are more symmetric for cMFs than they are for sMFs. Finally, once we turn on the magnetic field, while we maintain the overall shape of the profiles and do not affect τ_*tb*_, we intensify the correlations. Furthermore, cross-correlations decay slower. This means that the external magnetic field makes the reorientation of cMFs with superparamagnetic monomers more periodic.

**Figure 8:**
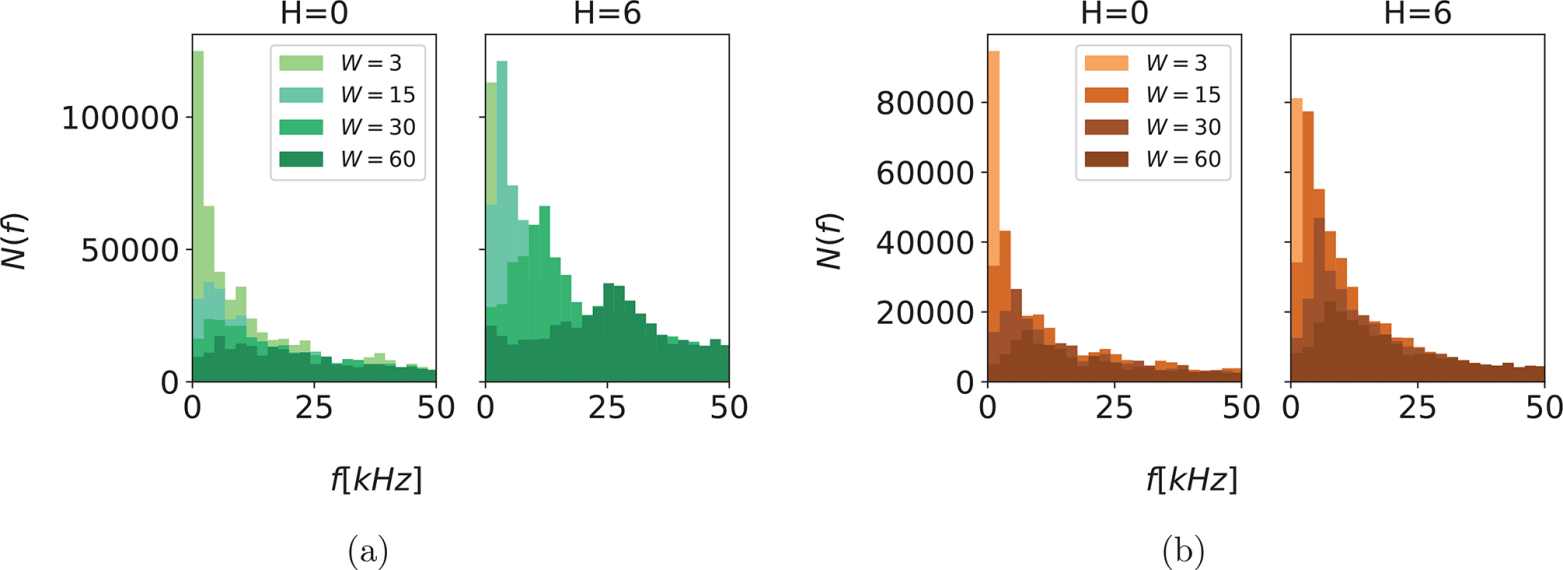
Frequency occurrence *N*(*f*) comparison between sMFs and cMFs with superparamagnetic monomers, for different *W*. (a) Results for sMFs in shear flow with (right; H = 6) and without (left; H = 0) an external magnetic field applied perpendicular to the flow direction. (b) Results for cMFs in shear flow with (right; H = 6) and without (left; H = 0) an external magnetic field applied perpendicular to the flow direction.

Further proof of our reasoning can be seen in the frequency spectrum of filament orientation with respect to the flow direction θ. In Figure 8, we show the occurrence of frequencies *N*(*f*), calculated by Fourier analysis of the time evolution of θ, between sMFs and cMFs with superparamagnetic monomers, for different *W*. While τ_*tb*_ estimated from *C*_*xz*_ gives us information about the frequency of the tumbling, *N*(*f*) tells us how frequent this tumbling is. Looking at Figure 8a, for sMFs we see that, with increasing *W*, *N*(*f*) shifts to higher frequencies. Without an external magnetic field applied, while there is a “preferred” frequency for a given value of *W*, the spectrum is rather smooth which suggests a lack of periodicity. Turning on an external magnetic field changes the *N*(*f*) distribution tremendously. For low shear *W* ∈ {3, 15}, preferred frequencies become more apparent but do not change substantially in comparison to the left figure. However, for higher *W* ∈ {30, 60} values, we see that the dominant frequencies change. This corresponds to collapsed conformations of MFs with superparamagnetic, spherical monomers, and the frequencies correspond to the oscillation eigenmodes of these globular structures that seem the be the dominant reorientational mode. The existence of collapsed conformations does not eliminate tumbling as such but is intertwined with it. Consequently, this complexity of the dynamics restricts how controllable sMFs with superparamagnetic monomers can be by magnetic fields in shear flow. As can be seen in Figure 8b, superparamagnetic, cubic monomers have a distinct advantage to this end. Without an external magnetic field applied, *N*(*f*) of preferred reorientational modes is slightly higher for cMFs than for sMFs, regardless of shear. Additional steric constraints imposed by monomer cubicity restrict the phase-space of accessible conformations. Turning on the external magnetic field makes the intensity of the preferred reorientational mode stand out even more. Cubic monomers preclude the possibility that the backbone collapses like it does for sMFs. For a strong magnetic field, the main driving mechanism of the reorientation is Zeeman coupling, which restricts the phase-space even more and increases the occurrence of tumbling.

## Conclusion

In this work, we used Molecular dynamics simulations coupled with the Lattice-Boltzmann method to understand how the magnetic nature and shape of monomers affects the dynamics of a single filament in shear flow with and without an external magnetic field applied perpendicular to the flow direction. To this end, we developed two computational models, namely, sMFs and cMFs, with spherical or cubic monomers, respectively, and constrained crosslinking. Furthermore, we considered monomers that represent two classes of MNPs: magnetizable, superparamagnetic MNPs and ferromagnetic ones. MFs in shear flow tumble, as is characteristic of polymer-like structures. Applying an external magnetic field perpendicular to the flow direction eliminates tumbling for MFs with ferromagnetic monomers and stabilizes the filament at a certain angle with respect to the flow direction. This angle is independent of monomer shape. cMFs stabilize in, on average, more *S*-shape conformations than sMFs, and their dipole moments are more aligned with the backbone than in sMFs.

Tumbling of a filament with superparamagnetic monomers can be eliminated with a magnetic field perpendicular to the flow direction only in low shear, where MFs can assume semipersistent, bent conformations. Outside of the low shear regime, MFs with superparamagnetic monomers tumble regardless of monomer shape. On average, MFs with cubic monomers are mostly aligned with the flow direction, while filaments with spherical monomers are not. Furthermore, for MFs with spherical monomers, frequency of tumbling changes with field strength in addition to shear strength. For cMFs this in not the case. The sMFs backbone collapses in strong shear flow with an external magnetic field applied. Such conformations are held together by magnetic interactions as well as entropy and have their own rotational eigenmodes. cMFs instead tumble in a distinct bend and flip motion. The occurrence of such motion can be enhanced by applying external magnetic fields. This investigation shows that MFs can achieve vastly different and systematically controllable behaviors.

A natural extension of this work would be to sample the free energy spectrum of cMFs that leads to dynamics we outlined in this work and investigate if and how these conclusions depend on filament length. Even though a suspension of MFs and concentration effects go beyond the present study, based on previous results obtained for self-assembly of cubic MNPs^86^ and static magnetization curves of MFs with cubic monomers,^80^ we expect the properties of a single cMF with superparamagnetic monomers presented in this work to be retained in a suspension of such filaments, up to concentrations where steric interactions dominate. On the other hand, brushes made of cMFs might be on average more compressible.^80^

## Simulation Methods

In this section we explain in detail the general computational scheme, interactions, and models used in this work.

### General Scheme

We performed Molecular dynamics (MD) simulations coupled with the Lattice-Boltzmann method^87,88^ in the ESPResSo software package.^89^ Particles in our simulations are propagated using time-discrete Newton’s equations of motion, integrated via the velocity Verlet algorithm.^90^ The excluded volume of each particle is achieved using the typical steric repulsion Weeks–Chandler–Andersen pair potential (WCA),:^91^
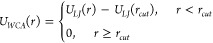
2where and *U*_*LJ*_(*r*) is the conventional Lennard–Jones potential:
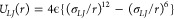
3where σ_*LJ*_ is the characteristic diameter of the particle and the cutoff value is *r*_*cut*_ = 2^1/6^σ. Parameter ϵ defines the energy scale of the repulsion.

We model the bonds as finitely extendable springs, described by the *FENE* potential:^92^
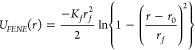
4where *K*_*f*_ is the rigidity of the bond, *r*_*f*_ is the maximal stretching length, and *r*_0_ is the equilibrium bond length. For sMFs, we place a FENE bond between the surfaces of each pair of neighboring monomers, so that when a filament is fully straight, the bonds are attached to the points where surfaces of neighboring particles would be touching. For cMFs, we capture the relevant characteristics of the intermonomer connections for *M*_*k*_^*l*^; *k* = 16 and/or *k* = 64 DNC from Xiong et al.^81^ Computationally, we realize this by attaching FENE bonds between adjacent corner particles on neighboring monomers, and between adjacent central edge particles on the faces of neighboring monomers.

The Lattice-Boltzmann method is an efficient grid-based hydrodynamics solver that is easily parallelizable and scalable. On the nanoscale, thermal fluctuations are relevant, and the simulated fluid must be thermalized. This can be achieved by adding stochastic fluctuations to the stress tensor, while conserving local mass and momentum conservation.^93−95^ Hydrodynamic forces are coupled to the MD scheme using a dissipative friction force, which also must be thermalized. The coupling force is given by^96^
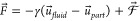
where γ is the friction parameter used to tune friction strength,  is the fluid velocity,  is the MD particle velocity, and  is the stochastic force respecting .

To be able to accurately capture the hydrodynamic effects on the monomers in our simulations, we used the so-called raspberry model.^97^ A single particle in a simulation box can only couple to a single lattice site per time step. Therefore, one cannot simulate the rotational diffusion of a single particle. Furthermore, the hydrodynamic impact of monomer shape would be neglected. However, using the raspberry model, one can construct a monomer with an arbitrary shape by homogeneously filling its volume with particles that serve as fluid coupling points. The MD scheme is coupled only to the center-of-mass particle, and all other particles in the monomer are fixed with respect to it. The moment of inertia tensor of this particle is set according to the mass and shape of the monomer. We constructed raspberry monomers for sMFs using the procedure described by Fischer et al.^98^ Cubic monomers in cMFs are modeled as a 5 × 5 × 5 mesh grid of MD particles. Construction of raspberry monomers to be used with the Lattice-Boltzmann method is a balancing act between computational load, friction, monomer shape, and how finely one needs to resolve its surface. Furthermore, particle grid size reflects on the time scales one can achieve in simulations. In general, particles filling out the volume of a raspberry monomer should be as homogeneously distributed as possible and their density, in conjunction with γ, must be tuned so that the translational and rotational diffusion coefficients *D*_*t*_ and *D*_*r*_ of the raspberry correspond to the expected hydrodynamic radius *r*_*h*_.

### Magnetic Interactions

Monomers in this work can be either ferromagnetic or superparamagnetic. Dipole moments of ferromagnetic monomers are modeled as central, point-particle dipole moments, *μ⃗*, of a fixed length , assigned to the center-of-mass particle of each monomer. Long-range magnetic interparticle interactions are accounted for via the standard dipole–dipole pair potential:
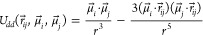
5where the intermonomer distance is , and  is the displacement vector connecting the *i* and *j* monomer center-of-mass with dipole moments  and , respectively. Zeeman interactions coming from the presence of an external magnetic field *H⃗* are realized via the Zeeman coupling potential:
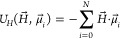
6To model the phenomenology of superparamagnetic MNPs accurately, we use the approach presented in Mostarac et al.^79^ One needs to calculate the total field  in each point of the system. The total magnetic field is the sum of *H⃗* and the dipole field . The latter field, created by magnetic particle *j*, at position  is given by
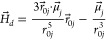
7

We define the dipole moment , of an *i*-th superparamagnetic particle at a given temperature *T*, as
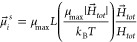
8where  is the modulus of the maximal magnetic moment, . Here, *k*_B_ is the Boltzmann constant and *L*(α) is the Langevin function:
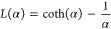
9Not only does this approach lend itself to account for nonlinear effects, but the expression 8 is a generalization of mean-field approaches, such as the modified mean field approach.^99^ The difference here is that we do not need to make any assumption to calculate . This approach is also verified by the analytical calculations for superparamagnetic particle magnetization.^100^

### Units

The interaction potential between a pair of monomers in our simulations is determined by the interplay between the steric interaction and the bonds between them. We match the parameters so that the magnitude of the interactions between nearest neighbors is nearly the same between sMFs and cMFs. The length scale in our simulations is prescribed by monomer hydrodynamic radius. The radius of monomers in our models corresponds to their hydrodynamic radius *r*_*h*_ = 9 nm. For sMFs this corresponds to a diameter σ = 3[*x*] in reduced units, where [*x*] is the length scale. For cMFs this corresponds to a cube side length σ = 2[*x*]. The simulation box is a 2D periodic rectangle with dimensions *L*_*box*_ = (140[*x*], 280[*x*], 280[*x*]), with periodic boundary conditions in the *y*–*z* plane.

The energy scale is the thermal energy in the system and is chosen to correspond to room temperature [*E*] = *k*_*b*_300*K*. Based on interaction potential matching, we determined that the energy scale of the steric repulsion between spherical monomers in sMFs should be achieved by a WCA potential on the center-of-mass particle corresponding to monomer size σ_*LJ*_ = 3 and ϵ = 1. Steric repulsion between cubic monomers of DNC MFs is achieved via a WCA potential on every particle on the surface on the monomer with σ_*LJ*_ = 0.5 and ϵ = 0.1.

Following the interaction potential matching strategy, we determined that FENE bonds in sMFs should be 9 times as rigid as the ones in cMFs. Therefore, *K*_*f*_ = 10 for cMFs, while *K*_*f*_ = 90 for sMFs. The equilibrium length of FENE bonds is set to be a multiple of monomer size *r*_0_ = 0.6σ. Maximal extension of each FENE bond, *r*_*f*_, was set to be 3 times the equilibrium bond length *r*_0_.

We choose the fluid density to correspond to water ρ_*w*_ = 1 × 10^3^ kg m^–3^. The LB grid spacing is set to *a*_*grid*_ = 1[*x*]. Therefore, the mass scale for sMFs is set to [*m*] = 2.15 × 10^–22^ kg, while for cMFs [*m*] = 7.3 × 10^–22^ kg. Time scales in our simulations are [*t*] = 1.37 × 10^–9^ s for sMFs and [*t*] = 3.78 × 10^–9^ s for cMFs. We set the fluid kinematic viscosity to ν = 0.1ν_*w*_ = 8.9 × 10^–8^ m^2^ s^–1^, corresponding to 3.4[*x*]^2^/[*t*] in simulation units for sMFs and 4.1[*x*]^2^/[*t*] for cMFs. This choice does not affect the physical results of our simulations while reducing the simulation time by an order or magnitude.

Monomers in our simulations correspond to Magnetite MNPs with a core density of , and a thin 1.5 nm oleic acid coating with . Therefore, the dimensionless dipolar coupling parameter between the monomers in our simulations is fixed to λ = 3. This also means that the maximum of the applied magnetic field range we explored represents strong fields of 0.26 T.

### Simulation Protocol

Characteristic relaxation time τ of a filament, which is defined as the time it takes for the autocorrelation function of the radius of gyration to decay in a thermalized fluid, is τ = 5.75 × 10^–6^. We adjusted the time step by which equations of motion propagate the system in all our simulations so that simulations elapse 8 × τ, regardless of model. Fluid flow lines were updated for each MD step. We create filaments of with *L* = 20 monomers and place each of them between two infinite planes at *x* = 0 and *x* = 140[*x*] of our 2D periodic rectangular simulation box with periodic boundary conditions in the *y*–*z* plane. Filament conformations are initially fully straight and stretched, with the backbone orientated randomly and placed in the center of the simulation box. We run 10 parallel simulations, for each (*W*, *H*) combination and filament model, at constant *T* = 1[*E*]. We place each filament in a thermalized fluid and let the fluid respond to the presence of the filament in the simulation box for 60 000 time steps. Afterward, we start the shear flow, by moving one of the planes with a given velocity with respect to the other. We measure every 6000 time steps 500 times.
